# Analysis of Keywords Used in Internet Searches for Melanoma Information: Observational Study

**DOI:** 10.2196/25720

**Published:** 2020-11-12

**Authors:** Japbani K Nanda, Jennifer L Hay, Michael A Marchetti

**Affiliations:** 1 Dermatology Service, Department of Medicine, Memorial Sloan Kettering Cancer Center, New York, NY, United States; 2 Department of Psychiatry and Behavioral Sciences, Memorial Sloan Kettering Cancer Center, New York, NY, United States

**Keywords:** internet, melanoma, skin cancer, health information, search, keyword, question, website, cost-per-click

## Abstract

**Background:**

The internet is an accessible resource for health care information and is often used by patients to learn about melanoma. The keywords that are used in internet searches can reflect internet users’ interest in specific topics and the public’s awareness of health-related issues.

**Objective:**

This study aims to describe the most frequently used keywords, questions, and corresponding websites in internet searches for melanoma.

**Methods:**

This is an observational study using data retrieved from Google Trends, Alexa Internet, SEMrush, Ahrefs, and SE Ranking for the keywords “melanoma” and “skin cancer.”

**Results:**

Average search interest as per Google Trends was greater for the keyword “skin cancer” than for the keyword “melanoma.” Searches for the top 25 keywords in 3 databases resulted in 34 unique melanoma keywords and 33 unique skin cancer keywords. Melanoma keywords were most frequently related to clinicopathologic classification (n=11, 32%), and skin cancer keywords were most frequently about diagnosis (n=14, 42%). Questions about the prognosis of melanoma appeared most frequently among the most popular melanoma questions, but general questions or questions about the diagnosis of melanoma contributed the greatest proportion of searches by search volume. Skin cancer question searches were most commonly about diagnosis. The highest proportion of searches for popular melanoma and skin cancer keywords most frequently sent traffic to websites from nonprofit organizations and media companies, respectively.

**Conclusions:**

We identified common keywords, questions, and websites used to access information about melanoma on the internet. These data may help health care providers and public health professionals when educating and counseling patients and the public about skin cancer.

## Introduction

In 2017, there were an estimated 1.2 million people living with invasive melanoma in the United States [1]. People may choose from a number of information sources to learn about melanoma, including the internet. In 2019, 90% of US adults used the internet, and 72% reported that they looked for medical or health information for themselves using a smartphone, computer, or other electronic means [2,3]. Although 95% of patients with melanoma at the University of Michigan reported that their physician provided sufficient information, 83% still looked to the internet for more information about melanoma [4]. Younger respondents, females, those with a higher level of education, and those diagnosed with melanoma were found to be more likely to search the internet for information about their diagnosis [4]. Although the majority of patients described their use of the internet as a positive experience, one-third of patients with melanoma have reported higher anxiety after internet use [4].

Researchers have previously studied trends in internet searches related to skin cancer. Using Google Trends, Bloom et al [5] found that internet searches for the terms “melanoma” and “skin cancer” in the United States increased each summer from 2010 to 2014. The peak for skin cancer searches occurred in May, which is skin cancer awareness month, suggesting a possible association with outreach programs [6]. German studies of Google search volume spanning 2013-2018 found that the most frequently used keywords for internet searches related to skin cancer were “skin cancer,” “white skin cancer,” “basalioma,” and “melanoma” [7,8]. Internet users searched for information about skin cancer types, identification, and skin changes [8]. The variety of search terms and questions used by internet users and the resulting websites accessed for information related to melanoma are not well characterized, particularly in the United States. Given the widespread use of the internet for health information broadly and skin cancer information specifically, our objective is to describe the keywords and questions used in melanoma internet searches. We also seek to identify the most frequently accessed websites for corresponding keyword searches.

## Methods

### Databases

This study was exempt from Institutional Review Board review because the data were publicly available. Data were obtained from multiple online databases: Google Trends [9], Alexa Internet [10], SEMrush [11], Ahrefs (URL Rating and Ahrefs Top) [12], and SE Ranking [13]. Google Trends provides keyword search interest data. Alexa, SEMrush, Ahrefs, and SE Ranking provide keyword or website analysis tools. Data from Alexa originates from the use of one of more than 25,000 browser extensions by millions of global internet users and from sites that install an Alexa script and permit Alexa to measure site traffic [14,15]. SEMrush uses third-party data providers to collect Google’s search results for the 500 million most popular keywords and collects information about websites in the top 100 positions [16]. Ahrefs uses independently obtained data across 10 different search engines (ie, does not use output from Alexa, Google, etc), processes clickstream data, and has the world’s largest third-party search query database [17,18]. SE Ranking is a cloud-based search engine optimization and marketing tool that tracks real-time website rankings for major search engines [19]. Data were extracted from databases on June 21, 2020, and analyzed using descriptive statistics.

### Keyword Search Interest and Search Volume

We defined a keyword as a term that is entered into a search engine (like Google), which subsequently lists websites on a results page. As internet users may initially search for information about melanoma with a more general term such as skin cancer, we performed searches and analyses for both melanoma and skin cancer. To determine search term interest (ranging from 0 to 100), we extracted US and worldwide web search data using Google Trends’ term comparison functionality to compare the terms “melanoma” and “skin cancer.” Search interest at a given time is presented relative to the peak popularity for a search term, which is indicated by a score of 100. We also recorded average monthly keyword search volumes over a 12-month period using SEMrush and Ahrefs.

### Keyword Popularity and Search Volume Analysis

We extracted the 25 most popular keywords related to melanoma and skin cancer searches using Alexa, SEMrush, and Ahrefs. For keywords that appeared in the first 25 consecutively listed keywords for one database but not another, the corresponding ranking in each database was recorded, if available. In Alexa, we determined the website with the highest share of voice for the keyword “melanoma.” Share of voice refers to the proportion of searches made for a keyword that result in traffic receipt by a specific website. Within the “Keyword Clusters” tool for organic keywords, we selected the melanoma cluster and ranked keywords by popularity (ranging from 0 to 100), which is updated monthly and indicates the relative frequency of searches for that keyword. In SEMrush, we searched for “melanoma” in the “Keyword Magic Tool” (US database) using the exact match filter, sorted by average monthly volume of searches during a 1-year period. In Ahrefs, we searched for “melanoma” in the “Keywords explorer” (Google, US database) using the phrase match filter, sorted by average monthly volume of searches. This process was similarly performed for the keyword “skin cancer” using Alexa, SEMrush, and Ahrefs. Keywords were classified into one of eight categories: general, diagnosis, clinicopathologic classification, etiology, prognosis, treatment, prevention, or screening.

### Keyword Advertising and Associated Websites

Keyword cost-per-click (CPC; USD) was recorded, if available, using SEMrush (based on Google Ads) and Ahrefs (based on multiple search engines). CPC is the average price paid by advertisers when their advertisement is clicked on in the results for that keyword. We recorded Alexa’s indicator of paid competition (ranging from 0 to 100), which is updated monthly and reflects the amount of advertisements that appear on major search engines for a searched keyword. We recorded the websites with the highest keyword share of voice per Alexa for each popular keyword. The organizations associated with popular websites were classified as nonprofit, media company, government, charity, or medical practice [20-35].

### Melanoma and Skin Cancer–Related Questions

The first 25 consecutively listed search questions related to melanoma and skin cancer were recorded using SEMrush’s “Keyword Magic Tool” (US database) and Ahrefs’s “Keywords explorer” (Google, US database). In SEMrush, questions were identified using the exact match filter and ranked based on average monthly volume of searches during a 1-year period. In Ahrefs, questions were ranked based on the average monthly volume of searches. Questions were classified into the same categories as keywords (previously mentioned).

### Popular Websites for Highest Ranked Melanoma and Skin Cancer Keywords

For the single most popular melanoma and skin cancer keywords, we used Alexa, Ahrefs, and SE Ranking to determine the 10 websites that received the highest share of voice of organic traffic or were the highest ranked. Alexa provided the websites with the highest share of voice during the prior 6 months. In Ahrefs, the websites were ranked by monthly estimated search traffic (Google, US). SE Ranking provided websites ranked on data from April 2020 (Google, US). For websites that appeared in the top 10 for one database and not another, the corresponding ranking for that website in each database was noted if available.

## Results

### Search Interest and Search Volume

“Skin cancer” was more frequently used than “melanoma” as a search term per Google Trends. From January 2004 to June 2020, average search term interest in the United States was 62 (min-max: 39-100) for “skin cancer” and 49 (min-max: 32-76) for “melanoma.” In June 2020 specifically, search term interest was 71 for “skin cancer” and 48 for “melanoma.” Worldwide, “skin cancer” was also more frequently used than “melanoma” (data not shown). The average US monthly search volume for the keyword “melanoma” was 246,000 (SEMrush) and 243,000 (Ahrefs); global estimates were 585,500 (SEMrush) and 644,000 (Ahrefs). The average US monthly search volume for the keyword “skin cancer” was 301,000 (SEMrush) and 190,000 (Ahrefs); global estimates were 547,300 (SEMrush) and 356,000 (Ahrefs).

### Popular Keywords

There were 34 unique keywords among the top 25 melanoma-related keywords in Alexa, SEMrush, and Ahrefs (Multimedia Appendix 1, Table S1). Keywords that appeared in the top 10 of all 3 databases were “melanoma,” “malignant melanoma,” “ocular melanoma,” “metastatic melanoma,” “melanoma symptoms,” “melanoma cancer,” “nodular melanoma,” and “melanoma pictures.” Keywords were most frequently related to melanoma clinicopathologic classification (n=11, 32%). There were 33 unique keywords among the top 25 skin cancer–related keywords in Alexa, SEMrush, and Ahrefs (Multimedia Appendix 1, Table S2). Keywords that appeared in the top 10 of all 3 databases were “skin cancer,” “skin cancer types,” “skin cancer pictures,” “what does skin cancer look like,” “skin cancer symptoms,” “skin cancer images,” and “signs of skin cancer.” Keywords were most frequently related to skin cancer diagnosis (n=14, 42%).

### Keyword Advertising and Commonly Accessed Websites

Using advertising competition data (paid competition and CPC) from multiple databases for the most popular keywords and questions in internet searches for melanoma and skin cancer, the keyword or question associated with the highest or one of the highest indicators of advertising competition (in the case of a tie) was related to treatment in 7 out of 10 cases. The keyword or question associated with the lowest indicator of advertising competition was related to diagnosis in 8 out of 10 cases.

A total of 11 unique websites had the highest share of voice per Alexa for the 34 melanoma keywords, and cancer.org (nonprofit; n=10, 29%) was the most frequent (Multimedia Appendix 1, Table S1). A total of 5 unique websites had the highest share of voice for the 33 skin cancer keywords, and webmd.com (media company; n=18, 55%) was the most frequent (Multimedia Appendix 1, Table S2).

### Popular Question Searches

The top 25 question searches about melanoma are shown in Multimedia Appendix 1, Table S3. Questions related to melanoma that appeared in the top 5 of SEMrush or Ahrefs were “what is melanoma,” “what does melanoma look like,” “does melanoma itch,” “what causes melanoma,” “what is melanoma cancer,” “what is the first sign of melanoma,” “what does early signs of melanoma look like,” and “where does melanoma spread to first.” Among the top 25 melanoma questions, the most frequent category was prognosis (16/50, 32%). Per SEMrush, general melanoma questions contributed the greatest proportion of searches by search volume (14,440/33,400, 43.2%). Per Ahrefs, questions about the diagnosis of melanoma contributed the greatest proportion of searches by search volume (16,200/41,100, 39.4%).

The top 25 question searches about skin cancer are shown in Multimedia Appendix 1, Table S4. Questions in the top 5 were “what does skin cancer look like,” “does skin cancer itch,” “is skin cancer itchy,” “can you die from skin cancer,” “is skin cancer deadly,” “how serious is basal cell skin cancer,” “what is skin cancer,” “what do the early stages of skin cancer look like,” and “what causes skin cancer.” Among the top 25 skin cancer questions, the most frequent category was diagnosis (27/50, 54%). Per SEMrush and Ahrefs, questions about diagnosis contributed the greatest proportion of searches by search volume (63,700/82,040, 77.6% and 30,400/43,400, 70.0%, respectively).

### Popular Websites for Highest Ranked Keywords

The 5 most popular or highest ranked websites for the keyword “melanoma” in Alexa, Ahrefs, and SE Ranking were skincancer.org (nonprofit), cancer.org (nonprofit), wikipedia.org (nonprofit), medicalnewstoday.com (media company), and mayoclinic.org (nonprofit). The 5 most popular or highest ranked websites for the keyword “skin cancer” were webmd.com (media company), skincancer.org (nonprofit), cancer.org (nonprofit), mayoclinic.org (nonprofit), and medicinenet.com (media company; Multimedia Appendix 1, Table S5).

## Discussion

### Principal Findings

Herein, we described the volume of searches, popular keyword search strategies, and popular websites accessed for information about melanoma and skin cancer on the internet. The most popular melanoma and skin cancer keywords were related to clinicopathologic classification and diagnosis, respectively. Queries about diagnosis often pertained to images, signs, or symptoms of skin cancer, suggesting a greater need for publicly available high-quality images of skin cancer. Our findings suggest that internet users are interested in online resources to learn about skin cancer diagnosis. This may also reflect awareness of the importance of skin cancer detection. Similarly, an analysis of internet search terms related to skin cancer in Germany found that keyword searches were often related to skin cancer identification via images or symptoms [7]. We also found that keyword searches related to prevention were uncommon. This may represent a need for increased awareness about the importance of primary prevention. Alternatively, this may suggest that the public already has adequate knowledge about preventative measures or that different, less technical terms are used, which were not captured in our study design.

With regard to searched questions, interest was directed toward melanoma prognosis, general information about melanoma, diagnosis of melanoma, and diagnosis of skin cancer. This may reflect internet users’ concerns regarding prognosis and the importance of prognostic and staging information in the determination of treatment options for melanoma. These findings further support the need for online resources with information about the detection of melanoma and skin cancer. Keyword searches related to treatment were less common for both melanoma and skin cancer. Our findings parallel the results of a German study that investigated topics of interest in internet searches related to melanoma and nonmelanoma skin cancer [8]. The study found that searches related to the treatment of skin cancer were made less frequently, while searches related to the forms of skin cancer were more common [8].

Our findings based on CPC data suggest that there is greater advertising competition for keywords related to the treatment of skin cancer than for the diagnosis of skin cancer. Nonprofit or charity-associated websites comprised 71% (24/34) and 36% (12/33) of the websites with the highest share of voice for popular melanoma and skin cancer keywords, respectively. Although cancer.org, a website of the nonprofit American Cancer Society [33], most frequently received traffic from the highest proportion of searches made for popular melanoma keywords, the highest proportion of searches for the great majority of popular skin cancer keywords sent traffic to webmd.com (WebMD LLC), which is a consumer health information brand under media company Internet Brands [31,32]. The type of website that receives a high share of traffic from internet searches may be affected by the specificity of a given keyword search as determined by the topic of interest, topic complexity, or the chosen keyword vocabulary. This is important to note because the quality and readability of health information can vary between websites associated with media companies, governmental organizations, and nonprofit groups [36-39].

An internet search for health information can affect treatment plans and serve as a topic of discussion between patients and health care providers. The majority (71%) of Canadian patients with melanoma found that information from the internet affected their decisions regarding treatment [40]. Most found the internet to be useful, albeit difficult to understand to various degrees [40]. Websites resulting from “melanoma” searches in 2017 demonstrated variability in quality and available content, as well as a higher than recommended reading level [41]. Therefore, physicians continue to play an important role by encouraging patients to discuss the findings of their internet searches for health information and addressing potential misinformation from online sources. Health care providers should be prepared to address questions or concerns that arise from patients’ health-related internet searches and guide patients toward reliable online sources of information. In this way, patients can better serve as active participants and advocates for their own health.

### Limitations

Our study is limited by its observational design using retrospective data and an inability to comment on the proportion of internet searches made by patients with or survivors of skin cancer or internet users at high risk for skin cancer. Nonetheless, our use of multiple keyword and website analysis services allowed us to evaluate the most popular keywords and websites accessed by the public. It is possible that internet users who searched for melanoma keywords may have been diagnosed with melanoma and sought to learn more about their diagnosis, while those who performed searches for skin cancer information may have been looking for general skin cancer information. A survey of 31 patients with melanoma found that 96% used the internet to acquire information about melanoma treatment [40]. Bloom et al [5] found a positive correlation between skin cancer search volume index and increased mortality due to melanoma, postulating that this may have been due to a higher volume of searches performed by individuals with advanced melanoma or others who were indirectly affected. Future research would be invaluable to study associations between the characteristics of internet users and specific keyword internet searches. Our study is also limited to analysis of the data as reported by keyword databases. Internet users can choose to learn about specific topics of interest or enhance their understanding of a given topic by directly clicking on links within an accessed webpage instead of performing a keyword search. Although keywords related to prevention were uncommon in our study, internet users may perform searches related to prevention using other keywords. For example, searches for sunburn prevention or sunscreen are relevant for skin cancer prevention but do not contain the keywords “melanoma” or “skin cancer.”

### Conclusions

Skin cancer is a public health issue that was highlighted in the US Surgeon General’s call for preventative action [42]. As a highly accessible resource, the internet is a valuable educational tool for skin cancer. Our study sheds light on internet users’ interests and awareness of topics related to melanoma and skin cancer. Our findings suggest that efforts should be made to ensure that the public has access to high quality information to address general concerns about melanoma and other interests related to the clinicopathologic classification, prognosis, and diagnosis of melanoma, as well as the diagnosis of skin cancer in general. This may also be an opportunity for public health professionals and clinicians to emphasize the importance of sun protective measures and skin cancer prevention. Public health professionals can create educational materials and initiate campaigns that effectively address topics of interest as expressed in internet searches while using keyword analysis to improve access to high quality resources. By understanding topics of interest, popular search queries, and frequently accessed websites for health information, clinicians can better prepare themselves to offer options for preferred information sources or critically evaluate the quality of online content accessed by patients. Familiarity with the types of skin cancer–related information that are of interest to the public and the most frequently accessed internet sources for this information may assist health care providers and public health professionals as they counsel and educate patients and the public on skin cancer.

## Supplementary Material

Multimedia Appendix 1

## Abbreviations

CPC: cost-per-click
