# Supplementary material for: Analysis of Keywords Used in Internet Searches for Melanoma Information: Observational Study
Source: JMIR Dermatol. Author manuscript; Available in PMC 2023 Mar 16. (PMC10018754; doi:10.2196/25720)
Supplement: Multimedia Appendix 1 [file NIHMS1828877-supplement-Multimedia_Appendix_1.docx]

## Multimedia Appendix

**Table S1** The 25 Most Popular Keywords in Internet Searches for Melanoma (Alexa, SEMrush, and Ahrefs)

|  | | **Alexa** | | | **SEMrush** | | **Ahrefs** | |
| --- | --- | --- | --- | --- | --- | --- | --- | --- |
| **Keyword** | **Keyword Type** | **Ranking (Popularity^a^)** | **Paid Competition^b^** | **Website with Most Share of Voice^c^ (%)** | **Ranking (Volume, thousands)** | **CPC^d^ (USD)** | **Ranking (Volume, thousands)** | **CPC^d^ (USD)** |
| melanoma | General | 1 (54) | 13 | skincancer.org (29.4) | 1 (246) | 2.1 | 1 (243) | 3.5 |
| malignant melanoma | General | 2 (35) | 18 | skincancer.org (21.0) | 5 (14.8) | 6.4 | 2 (18) | 7.0 |
| melanoma cancer pictures | Diagnosis | 3 (35) | 36 | webmd.com (47.3) | 3 (18.1) | 0.9 | >50 (0.3) | 0.7 |
| ocular melanoma | Clinicopathologic classification | 4 (34) | 12 | mayoclinic.org (21.4) | 10 (9.9) | 2.3 | 9 (8.7) | 2.5 |
| metastatic melanoma | Prognosis | 5 (34) | 15 | webmd.com (34.8) | 7 (12.1) | 12.3 | 10 (7.4) | 16 |
| melanoma symptoms | Diagnosis | 6 (33) | 34 | cancer.org (39.5) | 2 (27.1) | 1.1 | 3 (17) | 1.9 |
| melanoma cancer | General | 7 (32) | 35 | cancer.org (46.7) | 6 (14.8) | 3.0 | 7 (12) | 4.5 |
| nodular melanoma | Clinicopathologic classification | 8 (32) | 6 | webmd.com (26.5) | 9 (9.9) | 4.0 | 5 (12) | 3.5 |
| melanoma pictures | Diagnosis | 9 (32) | 39 | mayoclinic.org (28.9) | 4 (18.1) | 0.9 | 4 (14) | 1.2 |
| melanoma in situ | Clinicopathologic classification | 10 (29) | 22 | texasoncology.com (22.1) | 17 (5.4) | 2.1 | 17 (4.4) | 1.7 |
| what does melanoma look like | Diagnosis | 11 (29) | 28 | cancer.gov (39.2) | 14 (8.1) | 1.1 | 11 (7) | 1.2 |
| acral lentiginous melanoma | Clinicopathologic classification | 12 (29) | 1 | wikipedia.org (45.0) | 23 (3.6) | 4.3 | 14 (5) | 10.0 |
| what is melanoma | General | 13 (28) | 12 | skincancer.org (29.9) | 11 (9.9) | 2.4 | 13 (6.1) | 3.5 |
| melanoma treatment | Treatment | 14 (28) | 54 | cancer.org (39.4) | 12 (8.1) | 14.6 | 8 (8.7) | 17.0 |
| melanoma skin cancer | General | 15 (27) | 28 | cancer.org (51.2) | 8 (9.9) | 3.1 | 6 (12) | 4.0 |
| uveal melanoma | Clinicopathologic classification | 16 (26) | 5 | wikipedia.org (46.0) | 39 (2.4) | 4.0 | 27 (3.1) | 4.5 |
| melanoma staging | Prognosis | 17 (26) | 38 | cancer.org (23.4) | N/A | N/A | >50 (1.6) | 2.5 |
| amelanotic melanoma | Clinicopathologic classification | 18 (26) | 5 | skincancer.net.au (18.5) | 19 (4.4) | 3.9 | 15 (4.8) | 2.5 |
| lentigo maligna melanoma | Clinicopathologic classification | 19 (25) | 2 | dermnetnz.org (23.0) | 41 (1.9) | 2.9 | 28 (3.0) | 2.5 |
| subungual melanoma | Clinicopathologic classification | 20 (25) | 2 | verywellhealth.com (23.5) | 22 (4.4) | 4.8 | 12 (6.8) | 3.0 |
| superficial spreading melanoma | Clinicopathologic classification | 21 (25) | 5 | healthline.com (32.6) | 46 (1.9) | 3.6 | 26 (3.2) | 4.5 |
| abcde melanoma | Diagnosis | 22 (24) | 4 | skincancer.org (25.0) | 18 (4.4) | 3.1 | 24 (3.2) | 2.0 |
| melanoma stages | Prognosis | 23 (23) | 44 | cancer.org (19.0) | 16 (6.6) | 4.0 | 23 (3.8) | 4.5 |
| pictures of melanoma | Diagnosis | 24 (23) | 39 | webmd.com (26.6) | N/A | N/A | 20 (4.2) | 1.0 |
| melanomas | General | 25 (23) | 9 | skincancer.org (19.2) | N/A | N/A | N/A | N/A |
| signs of melanoma | Diagnosis | 27 (23) | 33 | skincancer.org (33.5) | 13 (8.1) | 1.7 | 21 (4.0) | 1.0 |
| melanoma mole | General | 30 (22) | 15 | skincancer.org (40.0) | 15 (6.6) | 1.4 | 22 (4.0) | 2.0 |
| eye melanoma | Clinicopathologic classification | >50 (16) | 15 | mayoclinic.org (35.6) | 20 (4.4) | 2.0 | >50 (1.0) | 2.5 |
| stage 4 melanoma | Prognosis | 26 (23) | 26 | healthline.com (59.0) | 21 (4.4) | 12.8 | 25 (3.2) | 7.0 |
| early melanoma | Prognosis | 39 (20) | 15 | cancer.org (41.1) | 24 (3.6) | 1.4 | 32 (2.8) | 2.5 |
| melanoma definition | General | 36 (20) | 2 | skincancer.org (21.0) | 25 (3.6) | 2.0 | 30 (2.9) | 2.5 |
| vulvar melanoma | Clinicopathologic classification | N/A | N/A | cancer.org (41.3) | 47 (1.9) | 2.1 | 16 (4.6) | 1.7 |
| what is the first sign of melanoma | Diagnosis | >50 (10) | 22 | cancer.org (50.5) | >50 (0.21) | 2.7 | 18 (4.2) | Not available |
| symptoms of melanoma | Diagnosis | 29 (22) | 25 | cancer.org (48.9) | N/A | N/A | 19 (4.2) | 1.8 |

^a^ Popularity (0-100) = updated each month and indicates the frequency of searches for that keyword; ^b^ Paid competition (0-100) = an indicator that is updated each month and reflects the amount of advertisements that appear on major search engines for a searched keyword; ^c^ Share of voice = proportion of searches made for a keyword that result in traffic receipt by a specific website; ^d^ CPC = cost-per-click, the average price paid by advertisers when their advertisement is clicked on in the results for that keyword; USD = United States dollars; N/A = not applicable

**Table S2** The 25 Most Popular Keywords in Internet Searches for Skin Cancer (Alexa, SEMrush, and Ahrefs)

|  | | **Alexa** | | | **SEMrush** | | **Ahrefs** | |
| --- | --- | --- | --- | --- | --- | --- | --- | --- |
| **Keyword** | **Keyword Type** | **Ranking (Popularity^a^)** | **Paid Competition^b^** | **Website with Most Share of Voice^c^ (%)** | **Ranking (Volume, thousands)** | **CPC^d^ (USD)** | **Ranking (Volume, thousands)** | **CPC^d^ (USD)** |
| skin cancer | General | 1 (49) | 30 | webmd.com (32.8) | 1 (301) | 2.6 | 1 (190) | 1.3 |
| types of skin cancer | Clinicopathologic classification | 2 (37) | 33 | webmd.com (33.9) | N/A | N/A | 2 (23) | 1.3 |
| skin cancer types | Clinicopathologic classification | 3 (37) | 44 | webmd.com (38.6) | 3 (49.5) | 0.9 | 3 (22) | 1.4 |
| skin cancer pictures | Diagnosis | 4 (35) | 44 | cancer.org (33.4) | 2 (49.5) | 0.3 | 6 (16) | 1.0 |
| what does skin cancer look like | Diagnosis | 5 (34) | 20 | webmd.com (47.1) | 5 (40.5) | 0.8 | 4 (22) | 1.6 |
| skin cancer symptoms | Diagnosis | 6 (34) | 20 | cancercenter.com (21.9) | 7 (33.1) | 1.5 | 7 (15) | 3.0 |
| skin cancer images | Diagnosis | 7 (33) | 31 | webmd.com (55.9) | 4 (40.5) | 1.1 | 5 (21) | 1.7 |
| signs of skin cancer | Diagnosis | 8 (30) | 32 | webmd.com (24.6) | 6 (33.1) | 1.1 | 9 (12) | 1.3 |
| skin cancer signs | Diagnosis | 9 (30) | 47 | webmd.com (29.5) | N/A | N/A | 11 (8.7) | 1.1 |
| symptoms of skin cancer | Diagnosis | 10 (29) | 22 | cancercenter.com (22.0) | N/A | N/A | 16 (5.8) | 0.9 |
| squamous cell skin cancer | Clinicopathologic classification | 11 (29) | 25 | skincancer.org (42.0) | 19 (8.1) | 1.3 | 15 (6.3) | 1.7 |
| skin cancer treatment | Treatment | 12 (28) | 33 | skincancer.org (25.6) | 18 (8.1) | 3.7 | 17 (5.5) | 6.0 |
| basal cell skin cancer | Clinicopathologic classification | 13 (28) | 20 | skincancer.org (41.8) | 13 (9.9) | 0.6 | 14 (7.1) | 1.7 |
| pictures of skin cancer | Diagnosis | 14 (28) | 38 | webmd.com (44.3) | N/A | N/A | 12 (7.8) | 0.9 |
| melanoma skin cancer | Clinicopathologic classification | 15 (27) | 28 | cancer.org (51.2) | 14 (9.9) | 3.1 | 8 (12) | 4.0 |
| skin cancer on face | Clinicopathologic classification | 16 (27) | 28 | webmd.com (41.7) | 8 (22.2) | 1.4 | 13 (7.6) | 1.8 |
| skin cancer on nose | Clinicopathologic classification | 17 (26) | 22 | webmd.com (37.8) | 12 (12.1) | 1.8 | 21 (3.5) | 1.7 |
| skin cancers | General | 18 (26) | 52 | webmd.com (23.9) | N/A | N/A | N/A | N/A |
| does skin cancer itch | Diagnosis | 19 (26) | 24 | webmd.com (44.9) | 20 (6.6) | 0.8 | 18 (4.6) | 1.3 |
| skin cancer screening | Screening | 20 (25) | 26 | aad.org (34.4) | >50 (1.9) | 3.1 | 39 (1.8) | 4.0 |
| skin cancer photos | Diagnosis | 21 (25) | 44 | cancer.org (35.7) | 11 (14.8) | 0.4 | 10 (11) | 1.1 |
| skin cancer spots | General | 22 (25) | 23 | webmd.com (54.5) | 15 (9.9) | 1.3 | 25 (3.0) | 1.3 |
| skin cancer moles | General | 23 (25) | 31 | cancer.org (27.9) | 10 (14.8) | 1.1 | 19 (4.2) | 1.7 |
| basal skin cancer | Clinicopathologic classification | 24 (24) | 16 | skincancer.org (45.3) | 23 (4.4) | 0.6 | 31 (2.2) | 1.7 |
| skin cancer pictures early stages | Diagnosis | 25 (24) | 36 | webmd.com (48.0) | 17 (8.1) | 0.6 | 20 (3.8) | 0.7 |
| dog skin cancer | Clinicopathologic classification | 28 (22) | 2 | webmd.com (52.2) | 9 (14.8) | 1.6 | 24 (3.0) | 2.0 |
| skin cancer on scalp | Clinicopathologic classification | 43 (18) | 24 | webmd.com (41.2) | 16 (8.1) | 2.6 | 26 (2.9) | 1.3 |
| early stage skin cancer | Prognosis | >50 (10) | 26 | webmd.com (48.4) | 21 (6.6) | 1.5 | >50 (0.35) | 2.0 |
| abcde skin cancer | Diagnosis | 35 (19) | 12 | skincancer.org (27.4) | 22 (5.4) | 2.8 | 40 (1.8) | 1.8 |
| early signs of skin cancer | Diagnosis | 45 (17) | 19 | webmd.com (44.5) | 24 (4.4) | 1.0 | 33 (2.1) | 1.5 |
| skin cancer bumps | General | >50 (7) | 12 | cancercenter.com (29.0) | 25 (4.4) | 3.7 | >50 (1.1) | 2.0 |
| skin cancer pics | Diagnosis | 40 (18) | 52 | cancer.org (38.6) | N/A | N/A | 22 (3.4) | 1.6 |
| how serious is basal cell skin cancer | Prognosis | N/A | N/A | skincancer.org (48.8) | 411 (0.26) | 1.6 | 23 (3.1) | Not available |

^a^ Popularity (0-100) = updated each month and indicates the frequency of searches for that keyword; ^b^ Paid competition (0-100) = an indicator that is updated each month and reflects the amount of advertisements that appear on major search engines for a searched keyword; ^c^ Share of voice = proportion of searches made for a keyword that result in traffic receipt by a specific website; ^d^ CPC = cost-per-click, the average price paid by advertisers when their advertisement is clicked on in the results for that keyword; USD = United States dollars; N/A = not applicable

**Table S3** The 25 Most Popular Melanoma Questions in Internet Searches (SEMrush and Ahrefs)

| **SEMrush** | | | | **Ahrefs** | | | |
| --- | --- | --- | --- | --- | --- | --- | --- |
| **Question** | **Question type** | **Ranking (Volume, thousands)** | **CPC^a^ (USD)** | **Question** | **Question type** | **Ranking (Volume, thousands)** | **CPC^a^ (USD)** |
| what is melanoma | General | 1 (9.9) | 2.4 | what does melanoma look like | Diagnosis | 1 (7) | 1.2 |
| what does melanoma look like | Diagnosis | 2 (8.1) | 1.1 | what is melanoma | General | 2 (6.1) | 3.5 |
| does melanoma itch | Diagnosis | 3 (1.6) | 1.4 | what is the first sign of melanoma | Diagnosis | 3 (4.2) | NA |
| what causes melanoma | Etiology | 4 (1.3) | 1.5 | what does early signs of melanoma look like | Diagnosis | 4 (2.4) | 0.7 |
| what is melanoma cancer | General | 5 (1.3) | 2.0 | where does melanoma spread to first | General | 5 (1.9) | 3.5 |
| what's melanoma | General | 6 (1.0) | 3.2 | what foods to avoid if you have melanoma | Prevention | 6 (1.9) | NA |
| is melanoma curable | Prognosis | 7 (0.88) | 3.2 | how long do you live after being diagnosed with melanoma | Prognosis | 7 (1.8) | NA |
| does melanoma hurt | Diagnosis | 8 (0.72) | 1.6 | what are the 4 types of melanoma | Clinicopathologic classification | 8 (1.8) | NA |
| how fast does melanoma spread | Prognosis | 9 (0.72) | 5.8 | how long does it take for melanoma to spread to organs | Prognosis | 9 (1.5) | NA |
| how to treat melanoma | Treatment | 10 (0.72) | 12.2 | how does melanoma make you feel | Diagnosis | 10 (1.4) | NA |
| how fast does melanoma grow | Prognosis | 11 (0.59) | 5.2 | what is the survival rate for melanoma | Prognosis | 11 (1.1) | 3.5 |
| is melanoma deadly | Prognosis | 12 (0.59) | 2.5 | how fast does melanoma spread | Prognosis | 12 (1.0) | 4.0 |
| is melanoma genetic | Etiology | 13 (0.59) | 2.6 | what causes melanoma | Etiology | 13 (1.0) | 3.0 |
| is melanoma hereditary | Etiology | 14 (0.59) | 2.1 | what does it mean to have a malignant melanoma | General | 14 (0.9) | NA |
| what does melanoma look like on the skin | Diagnosis | 15 (0.59) | 0.9 | how many people die from melanoma | Prognosis | 15 (0.8) | NA |
| what is malignant melanoma | General | 16 (0.59) | 4.1 | what is the most common treatment for melanoma | Treatment | 16 (0.8) | NA |
| how common is melanoma | General | 17 (0.48) | 3.5 | what is the survival rate for melanoma in the lymph nodes | Prognosis | 17 (0.7) | NA |
| how long does it take for melanoma to spread | Prognosis | 18 (0.48) | 7.1 | what is the latest treatment for melanoma | Treatment | 18 (0.6) | NA |
| can you die from melanoma | Prognosis | 19 (0.39) | 3.6 | what does melanoma look like in early stages | Diagnosis | 19 (0.6) | 0.2 |
| how do you get melanoma | Etiology | 20 (0.39) | 2.0 | who is most at risk for melanoma | General | 20 (0.6) | NA |
| how does melanoma spread | General | 21 (0.39) | 3.2 | what are the first signs of melanoma | Diagnosis | 21 (0.6) | NA |
| is melanoma cancer | General | 22 (0.39) | 4.0 | how long does it take for melanoma to spread | Prognosis | 22 (0.6) | 4.5 |
| is melanoma painful | Diagnosis | 23 (0.39) | 1.9 | how long can you live with untreated melanoma | Prognosis | 23 (0.6) | NA |
| what is metastatic melanoma | General | 24 (0.39) | 12.2 | what is the survival rate for malignant melanoma | Prognosis | 24 (0.6) | NA |
| can melanoma be cured | Prognosis | 25 (0.32) | 4.4 | what percentage of choroidal nevus becomes melanoma | General | 25 (0.6) | NA |

^a^ CPC = cost-per-click, the average price paid by advertisers when their advertisement is clicked on in the results for that keyword; USD = United States dollars; NA = not available

**Table S4** The 25 Most Popular Skin Cancer Questions in Internet Searches (SEMrush and Ahrefs)

| **SEMrush** | | | | **Ahrefs** | | | |
| --- | --- | --- | --- | --- | --- | --- | --- |
| **Question** | **Question type** | **Ranking (Volume, thousands)** | **CPC^a^ (USD)** | **Question** | **Question type** | **Ranking (Volume, thousands)** | **CPC^a^ (USD)** |
| what does skin cancer look like | Diagnosis | 1 (40.5) | 0.8 | what does skin cancer look like | Diagnosis | 1 (22) | 1.6 |
| does skin cancer itch | Diagnosis | 2 (6.6) | 0.8 | how serious is basal cell skin cancer | Prognosis | 2 (3.1) | NA |
| is skin cancer itchy | Diagnosis | 3 (3.6) | 0.8 | what is skin cancer | General | 3 (2.1) | 2.0 |
| can you die from skin cancer | Prognosis | 4 (2.9) | 1.6 | what do the early stages of skin cancer look like | Diagnosis | 4 (2.0) | NA |
| is skin cancer deadly | Prognosis | 5 (2.9) | 1.0 | what causes skin cancer | Etiology | 5 (1.6) | 1.9 |
| what is skin cancer | General | 6 (2.9) | 1.9 | how did bob marley die from skin cancer | General | 6 (1.5) | NA |
| what causes skin cancer | Etiology | 7 (2.4) | 1.1 | how to prevent skin cancer | Prevention | 7 (1.1) | 2.0 |
| what skin cancer looks like | Diagnosis | 8 (2.4) | 0.9 | what skin cancer looks like when it starts | Diagnosis | 8 (1.1) | 0.7 |
| does skin cancer hurt | Diagnosis | 9 (1.9) | 1.1 | how can you tell if a spot is skin cancer | Diagnosis | 9 (0.6) | NA |
| can skin cancer kill you | Prognosis | 10 (1.3) | 2.7 | what does skin cancer look like on a dog | Diagnosis | 10 (0.6) | 1.2 |
| is skin cancer curable | Prognosis | 11 (1.3) | 1.6 | how to tell if you have skin cancer | Diagnosis | 11 (0.6) | 1.7 |
| what does skin cancer on the face look like | Diagnosis | 12 (1.3) | 0.6 | how common is skin cancer | General | 12 (0.6) | 3.0 |
| how do you get skin cancer | Etiology | 13 (1.0) | 1.1 | what does skin cancer feel like | Diagnosis | 13 (0.6) | 2.0 |
| how do you know if you have skin cancer | Diagnosis | 14 (1.0) | 0.8 | what can you eat to prevent skin cancer | Prevention | 14 (0.6) | NA |
| how does skin cancer look | Diagnosis | 15 (1.0) | 1.1 | how do you know if a spot is skin cancer | Diagnosis | 15 (0.6) | NA |
| how to know if you have skin cancer | Diagnosis | 16 (1.0) | 0.9 | what does skin cancer look like? | Diagnosis | 16 (0.5) | NA |
| how to treat skin cancer | Treatment | 17 (1.0) | 6.7 | how long can you live with skin cancer | Prognosis | 17 (0.5) | NA |
| can dogs get skin cancer | General | 18 (0.88) | 4.9 | at what age does skin cancer typically occur | General | 18 (0.5) | 4.5 |
| can skin cancer itch | Diagnosis | 19 (0.88) | 0.9 | how does skin cancer start | Etiology | 19 (0.5) | 1.6 |
| how to prevent skin cancer | Prevention | 20 (0.88) | 1.8 | what is the most common type of skin cancer | Clinicopathologic classification | 20 (0.45) | 6.0 |
| how to spot skin cancer | Diagnosis | 21 (0.88) | 2.5 | what skin cancer looks like | Diagnosis | 21 (0.45) | 1.5 |
| how to tell if you have skin cancer | Diagnosis | 22 (0.88) | 0.8 | how to spot skin cancer | Diagnosis | 22 (0.45) | 1.2 |
| is skin cancer genetic | Etiology | 23 (0.88) | 2.8 | how is skin cancer treated | Treatment | 23 (0.45) | 3.5 |
| what are the signs of skin cancer | Diagnosis | 24 (0.88) | 0.9 | what does the beginning of skin cancer look like | Diagnosis | 24 (0.45) | 1.9 |
| what does skin cancer look like on a dog | Diagnosis | 25 (0.88) | 0.9 | what does skin cancer look like on your face | Diagnosis | 25 (0.45) | 1.4 |

^a^ CPC = cost-per-click, the average price paid by advertisers when their advertisement is clicked on in the results for that keyword; USD = United States dollars; NA = not available

**Table S5** Most Popular or Highest Ranked Websites for Keywords “Melanoma” and “Skin Cancer”

| **Keyword: “Melanoma”** | | | | **Keyword: “Skin Cancer”** | | | |
| --- | --- | --- | --- | --- | --- | --- | --- |
| **Website** | **Alexa Ranking (Share of voice, %)^a^** | **Ahrefs Ranking** | **SE Ranking Ranking** | **Website** | **Alexa Ranking (Share of voice, %)^a^** | **Ahrefs Ranking** | **SE Ranking Ranking** |
| skincancer.org | 1 (29.4) | 3, 4 | 2, 4 | webmd.com | 1 (32.8) | 1 | 2 |
| cancer.org | 2 (14.4) | 5 | 5 | skincancer.org | 2 (22.1) | 4 | 3, 4 |
| wikipedia.org | 3 (13.5) | N/A | 7 | cancer.org | 3 (10.9) | 6, 7 | 6, 7 |
| medicalnewstoday.com | 4 (7.1) | 6 | 6 | mayoclinic.org | 4 (9.5) | 3 | 1 |
| mayoclinic.org | 5 (6.7) | 2 | 3 | medicinenet.com | 5 (6.7) | 5 | 5 |
| webmd.com | 6 (5.2) | 7 | 11 | aad.org | 6 (3.0) | 8 | 8 |
| macmillan.org.uk | 7 (4.4) | 63 | N/A | emedicinehealth.com | 7 (2.6) | N/A | 21 |
| cancer.net | 8 (3.7) | 19 | 16 | wikipedia.org | 8 (1.6) | 20 | 20 |
| healthline.com | 9 (2.5) | 16 | 18 | medicalnewstoday.com | 9 (1.3) | 15 | 12 |
| medlineplus.gov | 10 (2.2) | 8 | 10 | healthline.com | 10 (1.2) | 9 | 10 |
| cancer.gov | 11 (1.4) | 9 | 9 | dermnetnz.org | 39 (0.02) | 10 | 13 |
| medicinenet.com | 14 (1.2) | 10 | 8 | medlineplus.gov | 11 (1.2) | 11 | 9 |

Based on share of voice in Alexa and URL ranking positions in Ahrefs and SE Ranking. Alexa rankings are based on share of voice. SE Ranking provided websites ranked on data from April 2020 (Google, US) and in Ahrefs, the websites were ranked by monthly estimated search traffic (Google, US). For websites that appeared in the top 10 for one database and not another, the corresponding ranking for that website in each database was noted if available. All rankings within the top 10 or the single highest ranking beyond the top 10 were included. For melanoma, there were no websites listed in the number 1 ranking position in Ahrefs and SE Ranking. For skin cancer, there was no website listed in the number 2 ranking position in Ahrefs.

N/A = not applicable

^a^ Share of voice = proportion of searches made for a keyword that result in traffic receipt by a specific website
